# The Use of Titanium 3D Mini-Plates in the Surgical Treatment of Fractures of the Mandibular Condyle: A Systematic Review and Meta-Analysis of Clinical Trials

**DOI:** 10.3390/jcm10163604

**Published:** 2021-08-16

**Authors:** Maciej Sikora, Maciej Chęciński, Zuzanna Nowak, Kamila Chęcińska, Tomasz Olszowski, Dariusz Chlubek

**Affiliations:** 1 Department of Maxillofacial Surgery, Hospital of the Ministry of Interior, Wojska Polskiego 51, 25-375 Kielce, Poland; sikora-maciej@wp.pl; 2 Department of Biochemistry and Medical Chemistry, Pomeranian Medical University, Powstańców Wielkopolskich 72, 70-111 Szczecin, Poland; 3 Preventive Medicine Center, Komorowskiego 12, 30-106 Kraków, Poland; maciej@checinscy.pl; 4 StomaDent Non-Public Healthcare Institution, Dental Clinic, Kościuszki 32, 46-320 Praszka, Poland; zuzannaewanowak@gmail.com; 5 Department of Glass Technology and Amorphous Coatings, Faculty of Materials Science and Ceramics, AGH University of Science and Technology, ul. Mickiewicza 30, 30-059 Kraków, Poland; kamila@checinscy.pl; 6 Department of Hygiene and Epidemiology, Pomeranian Medical University, Powstańców Wielkopolskich 72, 70-111 Szczecin, Poland; tomasz.olszowski@pum.edu.pl

**Keywords:** mandibular condyle fracture, osteosynthesis, open reduction and internal fixation, fracture fixation, miniplate, three-dimensional plate

## Abstract

Introduction: Fixing fractures of the base and neck of mandibular condyles is demanding due to the difficulties in surgical access and the various shapes of bone fragments. Classic fixation techniques assume the use of straight mini-plates, utilized for other craniofacial bone fractures. Three dimensional mini-plates may provide a reasonable alternative due to their ease of use and steadily improved mechanical properties. The multitude of different shapes of 3D mini-plates proves the need for their evaluation. Aim: This paper aims to summarize the clinical trials regarding the use of various types of 3D condylar mini-plates in terms of need for reoperation and the incidence of loosening and damage to the osteosynthetic material. Materials and Methods: A systematic review was conducted in accordance with PICOS criteria and PRISMA protocol. The risk of bias was assessed using ROBINS-I and RoB 2 Cochrane protocols. The obtained data series was analyzed for correlations (Pearson’s *r*) respecting statistical significance (Student’s *t*-test *p* > 0.05) and visualized using OriginLab. Results: 13 clinical trials with low overall risk of bias regarding 6 shapes of 3D mini-plates were included in the synthesis. The number of reoperations correlates with the number of fixations (*r* = 0.53; *p* = 0.015) and the total number of screw holes in the mini-plate (*r* = −0.45; *p* = 0.006). There is a strong correlation between the number of loosened osteosynthetic screws and the total number of fractures treated with 3D mini-plates (*r* = 0.79; *p* = 0.001 for each study and *r* = 0.99; *p* = 0.015 for each mini-plate shape). A correlation between the percentage of lost screws and the number of distal screw holes is weak regarding individual studies (*r* = −0.27; *p* = 0.000) and strong regarding individual mini-plate shape (*r* = −0.82; *p* = 0.001). Three cases of 3D mini-plate fractures are noted, which account for 0.7% of all analyzed fixation cases. Discussion: The reasons for reoperations indicated by the authors of the analyzed articles were: mispositioning of the bone fragments, lack of bone fragment union, secondary dislocation, and hematoma. The known screw loosening factors were poor bone quality, bilateral condylar fractures, difficulties in the correct positioning of the osteosynthetic material due to the limitations of the surgical approach, fracture line pattern, including the presence of intermediate fragments, and mechanical overload. Fractures of the straight mini-plates fixing the mandibular condyles amounts for up to 16% of cases in the reference articles. Conclusions: There is no convincing data that the number of reoperations depends on the type of 3D mini-plate used. The frequency of osteosynthetic screw loosening does not seem to depend on the 3D mini-plate’s shape. Clinical fractures of 3D mini-plates are extremely rare.

## 1. Introduction

Fixing fractures of the mandibular condyles is problematic due to the difficulties in surgical access [1,2,3]. The open view into the fracture fissure is guarded by the skin of an aesthetic preaural area and a very delicate facial nerve [1,2,3]. The difficulties in surgical access can be partially overcome by using surgical techniques adequate to the fracture’s height [2,3,4]. However, a crucial step of fixing two or more bone fragments of the neck and the base of the mandibular condyle is another issue [5,6,7]. The still modernized classifications dividing the fractures of the mandibular condyle depending on the height of the fissure and distinguishing squat and slender condyles can be helpful [4,5]. Despite the ongoing evaluation of operational techniques, no consensus has been reached on the type and shape of the fixation material [6,8,9]. There are known ways to fix condyles with screws, but mini-plates dominate around the base and the lower part of the neck [6,7,8,10]. While classic techniques assume the use of straight mini-plates, used for fixation of other craniofacial bones, recent years have confirmed the popularity of 3D mini-plates with dedicated shapes [6,7,8]. Some of these condylar mini-plates even come in right and left variants to best fit the bone fragments of a particular side [6,8]. The multitude of different solutions proves the search for a gold standard [6,8,11]. Recent reports focus on an adequate number of screws in the proximal bone fragment and the possibility of fixation of intermediate fragments using the single-plate technique [6,8]. In vitro studies conducted on human macrophages derived from THP-1 cells show that the presence of 3D mini-plates does not increase the inflammatory response, therefore confirming the implants’ safety [12,13,14]. The in vitro strength testing of 3D mini-plates also show promising results [8,10,13,14,15,16,17,18].

## 2. Aim

This paper aims to summarize clinical trials regarding the use of various types of 3D condylar mini-plates. The specific objectives set out by the authors of this paper were to assess the fixation of the mandibular condyles with various types of individual 3D mini-plates in terms of the need for reoperation and the incidence of loosening and damage to the osteosynthetic material.

## 3. Materials and Methods

### 3.1. Eligibility Criteria

The above-mentioned goal was achieved by designing and conducting a systematic review with meta-analysis of the results. In the first stage, two authors (M.C. and Z.N.) defined the eligibility criteria for the research results presented in the articles of other authors. For this purpose, the PICOS criteria were adapted and implemented, taking into account 5 aspects corresponding to the extensions of this acronym [19]. (1) Population: in relation to the study population, the diagnosis of base or neck fractures of the mandibular condyle was accepted, thus rejecting the fractures of the mandibular head [20]. Only human patients were considered. (2) Intervention: the required surgical intervention was a stable osteosynthesis treatment method carried out with the use of a single 3D mini-plate. Thus, studies involving the use of straight mini-plates or techniques allowing the mixed use of a 3D mini-plate and any other form of fixation (e.g., additional 3D mini-plate, straight mini-plate, or lag screw) were rejected. (3) Comparison: no control group was required; however, the level of evidence was assessed, taking into account, among other things, the presence of a control group, e.g., those treated surgically with another technique or treated conservatively. It was decided not to exclude studies due to any type of comparison being conducted. (4) Outcomes: reporting of complications in the form of reoperation, screw loosening or damage to the 3D mini-plates was needed. No criterion was adopted to exclude articles based on treatment outcomes. (5) Study design: due to the high expectations of the authors of this paper in regard to its future usefulness, it was decided to reject studies with evidence levels 4 and 5, i.e., based on case series, single cases, or not supported by clinical material. For this purpose, a positive criterion was adopted for the inclusion of only designed studies, i.e., experimental and observational (both prospective and retrospective), carried out on at least 10 cases. For the same reasons, research with moderate or higher risk of bias, papers published before 2010, articles not available in English, and those non-indexed in the PubMed medical articles database (32 million records) were rejected. The authors of this systematic review concluded that PubMed indexing constitutes additional indirect evidence of the reliability of the studies that were compiled in the course of this systematic review. Briefly, the eligibility criteria are presented in Table 1.

### 3.2. Search Strategy

The applied search strategy was based on predefined PICOS criteria [19]. This strategy was initially developed and repeatedly improved by two of the authors (M.C. and Z.N.), giving the final result in the form presented by the following formula: (mandible OR lower jaw) AND (condyle OR condyles OR condylar OR base OR neck OR cervix OR collum) AND (fracture OR fractures) AND (3D OR 3-dimensional OR three-dimensional OR multidimensional OR multi-dimensional OR strut OR rhombic OR trapezoid OR TCP OR delta OR DCCP OR lambda OR A-shape OR ACP OR X-shape OR XCP OR grid OR matrix) AND (plate OR plates OR miniplate OR miniplates OR mini-plate OR mini-plates).

The search strategy presented above allowed the authors to specify at this stage the detailed data on the study group (Population) and its treatment (Intervention) in relation to the positive criteria.

### 3.3. Exclusion Protocol

The search of medical databases presented results in the form of records containing specific numbers (i.e., DOI and PMID), titles, and names of authors of individual articles. These data were exported to the Rayyan QCRI application. This application made it possible to view abstracts and basic data on articles, and thus to carry out the next stages of excluding studies that do not meet the PICOS criteria [19]. Subsequent stages of trial selection were carried out in accordance with the PRISMA protocol, consisting of the stages of identification, screening, and eligibility [21]. (1) Identification: the identification step was performed by two authors (M.C. and Z.N.) based on the above-described search strategy. Due to high expectations regarding the quality of qualified research, attempts to identify additional articles in gray literature were abandoned. (2) Screening: analyses of the found articles’ abstracts were completed by the same two authors (M.C. and Z.N.). The convergence of assessments at the screening stage was determined using Cohen’s kappa coefficient (*k* = 0.968). In cases of conflict at the screening stage, an article was considered, included, and processed to the next stage, i.e., eligibility. (3) Eligibility: the final assessment regarding the acceptance of studies for meta-analysis or rejection was made by three authors (M.S., M.C. and Z.N.) based on the full texts of articles. At this stage, the authors’ assessments regarding qualifications were fully consistent.

### 3.4. Qualitative Assessment

The quality of the individual studies described in the eligible articles was assessed by two authors (M.C. and Z.N.) by determining the level of evidence and risk of bias. In accordance with the previously adopted study design criterion, only articles with the following levels of evidence were included: (1) randomized controlled trials, (2) cohort studies, and (3) case control studies. All the studies included discuss in their entirety the clinical trials assuring the high evidence level of the following systematic review. The risk of bias in non-randomized trials was determined via the Cochrane ROBINS-I protocol and for randomized trials via the Cochrane RoB 2 protocol [22,23]. The risk of bias was assessed with regard to those aspects of each study that were relevant to the meta-analysis contained in this paper. In assessing the risk of bias, a simplified scale was used with the following numeric values: (1) Low, (2) Moderate, and (3) Serious. The overall risk of bias in each trial was calculated as the arithmetic mean of the risks for the individual domains and was expressed on the same 3-point scale with values from 1 to 3, where 3 represented the highest risk of bias.

### 3.5. Data Extraction and Synthesis

From the included articles, two authors (M.C. and K.C.) extracted data of a qualitative (3D mini-plate type) and quantitative nature. These data are tabulated in the text of this article. The quantitative data concerned: (1) the number of proximal and distal holes in the different types of 3D mini-plates used; (2) the number of mandibular condyles fixed using 3D mini-plates; (3) numbers and rates of complications, including: (a) need for reoperation; (b) loosening of one or more screws; (c) fracture of the mini-plate. The obtained and compiled data were analyzed for correlation using Pearson’s correlation coefficient and Student’s *t*-test and visualized using OriginLab (Northampton, MA, USA). The results were interpreted by 4 authors (M.S., M.C., K.C. and D.C.).

## 4. Results

As a result of the medical databases search performed on April 10, 2021, 165 unique records corresponding to the relevant articles were identified. During the screening phase, 147 articles were excluded, which accounted for 89.1% of the initially identified records. The reasons for excluding articles according to the PICOS acronym were as follows: (1) inappropriate study design (S = 88); (2) wrong study group (P = 56); (3) treatment other than expected (I = 3) [19]. In cases where there were several reasons for excluding a single article, only one of them was taken into account, in the order presented above. At the screening stage, no articles were excluded due to inadequate controls (C = 0) or adverse treatment outcomes (O = 0) [19]. Thus, 18 full-text articles were included in the eligibility stage.

Further exclusion of articles at the eligibility stage resulted in the rejection of another 9 entries. Among them, five studies were disqualified due to inadequate study design (S = 5), three were carried out on inadequate study groups (P = 3) and one presented invalid intervention (I = 1). Out of 9 articles included, 2 reported two groups of patients and 1 reported three groups of patients, each of whom had been treated with specific types of 3D mini-plates. Thus, 9 articles describing a total of 13 clinical trials on the clinical use of 3D mini-plates were included in the synthesis.

Seven of the eligible articles reported nonrandomized studies and underwent a bias risk assessment protocol called ROBINS-I [22]. The remaining 2 articles describing randomized trials were assessed for the risk of bias using a dedicated RoB 2 [23] algorithm. The individual domain scores assessed in both protocols for clarity are summarized in Table 2. Summary scores for each of the included articles indicated a low risk of bias. Therefore, no study was rejected due to an excessively high risk of bias and all of them proceed to the meta-analysis.

The combined results of the studies described in the articles included in the meta-analysis concerned 6 different shapes of 3D mini-plates, i.e., trapezoid, deltoid, rhombus, strut, 9-hole trapezoid, and lambda (Table 3). In total, 455 cases of mandibular condyle fractures treated with the above-mentioned shapes of 3D mini-plates were included in the meta-analysis. Of these, 10 cases (2.2%) required reoperation, in 14 cases (3.0%) at least one of the osteosynthetic screws loosened, and in 3 cases (0.7%) the 3D mini-plate was broken.

Trapezoidal 3D mini-plates were evaluated in 5 studies with varying levels of evidence, including a total of 150 fracture cases. Among them, there were 6 cases (4%) of loosening of osteosynthetic screws, 3 cases (2%) requiring reoperation, and a single case (0.7%) of fracture of the 3D mini-plate. The evaluation of the deltoidal 3D mini-plates was based on the results of 4 studies, with levels of evidence from 1 to 3, involving a total of 140 fracture cases. In 5 of these cases (3.6%) concerning delta mini-plates one or more osteosynthetic screws were found to be loose and single (0.7%) mini-plate fracture and one (0.7%) reoperation were noted.

The remaining 3D mini-plates were assessed based on retrospective observational case-control studies. 3D rhombic mini-plates were used in one of the qualified studies, carried out on a group of 92 fracture cases. Reoperation was required in 6 cases (6.5%), and in 3 cases (3.3%) the screws for osteosynthesis were loosened. However, no fracture of a single rhombus-shaped plate was found. The strut, 9-hole trapezoidal and lambda 3D mini-plates were evaluated from single studies on groups of 34, 28 and 11 fractures, respectively. One strut mini-plate was fractured, and no other failures of these types of osteosynthetic material or reoperations were found. Detailed data are presented in Table 4.

A search for correlation between the numerical data was carried out. Correlation matrices are tabulated for individual studies (Table 5) and for case sums for each type of 3D mini-plate (Table 6).

Attempts to search for dependencies expressed by the Pearson correlation coefficient between the number of reoperations and the remaining numerical data presented statistically insignificant results in the context of individual studies by various authors. For the sums of the application cases of individual shapes of 3D mini-plates, the correlations of the number of reoperations with the number of fixations (*r* = 0.53; *p* = 0.015) and the total number of screw holes in a given type of 3D mini-plate (*r* = −0.45; *p* = 0.006) are statistically significant. These correlations have an absolute value close to half (|*r*| = 0.5), which means that they are on the border of strong and weak correlations. Thus, it should be assumed that the number of reoperations may be somewhat directly dependent on the number of operations in general and at the same time may be somewhat inversely dependent on the number of holes in the 3D mini-plate, and thus on the number of screws used in osteosynthesis. It should be emphasized that there are no statistically significant correlations between the number of reoperations and the number of loosened osteosynthetic screws, both in relation to individual clinical trials and in relation to the total cases for specific shapes of 3D mini-plates.

**Figure 1:**
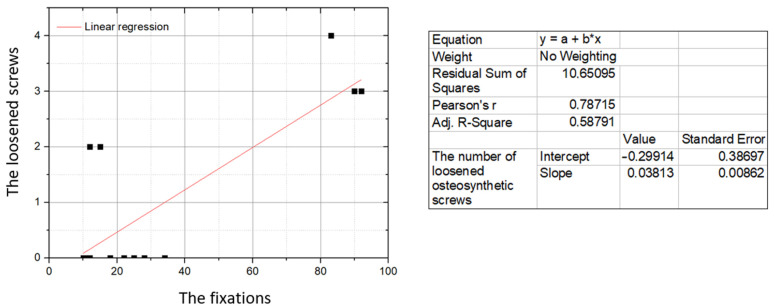
Correlation between the number of loosened osteosynthetic screws (vertical axis) and the number of fixations with 3D mini-plates (horizontal axis). Data for individual studies.

**Figure 2:**
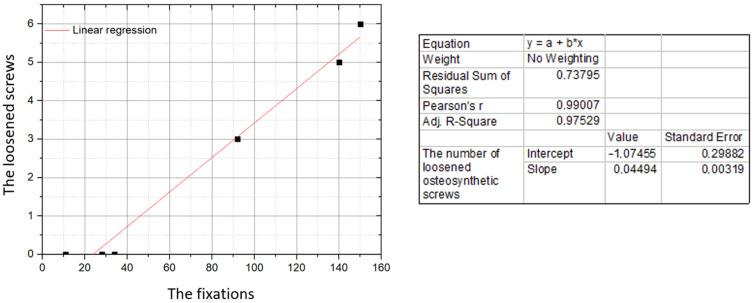
Correlation between the number of loosened osteosynthetic screws (vertical axis) and the number of fixations with 3D mini-plates (horizontal axis). Data for individual shapes of 3D mini-plates.

There is a strong positive correlation between the number of loosened osteosynthetic screws and the total number of fractures treated with 3D mini-plates (*r* = 0.79; *p* = 0.001; Figure 1). For the total numbers of cases sorted according to the shapes of the 3D mini-plates, the Pearson correlation coefficient is close to one (*r* = 0.99; *p* = 0.015; Figure 2). Therefore, the total number of complications in the form of loosening screws in the study material is nearly directly proportional to the total number of fixed fractures, which may indirectly prove that the cases of loosening of the osteosynthetic screws are independent of the shape of the 3D mini-plate. The percentage of loose osteosynthetic screws for the entire material tested was 3.08%.

**Figure 3:**
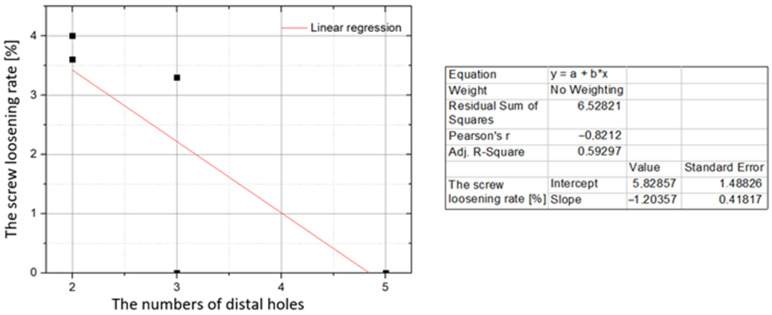
Correlation between the screw loosening rate (vertical axis) and the distal number of holes in the 3D mini-plate (horizontal axis). Data for individual studies.

**Figure 4:**
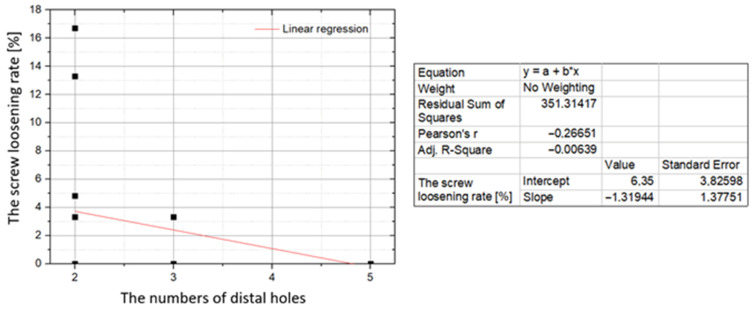
Correlation between the screw loosening rate (vertical axis) and the number of distal holes in the 3D mini-plate (horizontal axis). Data for individual shapes of 3D mini-plates.

In the analysis of clinical trials, a weak negative correlation between the percentage of lost screws and the number of distal screw holes in each study was observed (r = −0.27; *p* = 0.000; Figure 3). The same type of correlation was found to be strong after summing the cases and analyzing the data for each type of 3D mini-plate (r = −0.82; *p* = 0.001; Figure 4).

The total number of holes for the osteosynthetic screws in the analyzed types of 3D mini-plates was between 4 to 9. The 9-hole Trapezoid mini-plate has 4 holes for the fixation of the proximal bone fragment. All other 3D mini-plates presented in the analyzed studies have 2 proximal holes. The total number of holes in a single 3D mini-plate is therefore primarily dependent on the number of holes for the distal bone fragment, which ranges from 2 to 5 depending on the type of mini-plate. The highest rates of osteosynthetic screw loss were observed for 3D mini-plates with only 4 holes (2 for proximal and distal parts). The proportions of screw loss were 4.00% and 3.57% for the two shapes of 4-hole 3D mini-plates, trapezoid and deltoid, respectively. In the analysis of clinical trials, a weak negative correlation between the percentage of lost screws and the number of distal screw holes in each study was observed (*r* = −0.27; *p* = 0.000). The same type of correlation was found to be strong after summing the cases and analyzing the data for each type of 3D mini-plate (*r* = −0.82; *p* = 0.001). Therefore, one should take into account the possibility of the loosening of osteosynthetic screws, resulting from the insufficiently tight fastening made with only 4 screws.

In all the examined material, 3 cases of fractures of 3D mini-plates were found. Each of these cases concerned a different shape of the 3D mini-plate: trapezoid, deltoid, and strut. Due to the extremely low number of fractures of the 3D mini-plates, no attempts were made to interpret the correlation values. Each 3D mini-plate fracture is discussed individually in the discussion of this article.

## 5. Discussion

Not all mandibular condyle fractures require fixation [1,2,3,33]. The guidelines for the classification of condylar fractures are currently under discussion and are constantly evolving [3,4,20]. Thus, non-displaced and slightly displaced fractures are qualified by numerous authors for conservative treatment [1,3,33]. In turn, displaced fractures shortening the mandibular branch or characterized by displacement of the bone fragments in axes other than vertical, especially with proximal bone fragments dislocated from within the temporomandibular joint, usually qualify for surgical treatment [1,3,33]. Surgical techniques include minimally invasive endoscopic treatment and more invasive open approaches [2,3,34]. The latter differ significantly depending on the location of the fracture gap [3,4,33]. Fractures of the mandibular head and neck are operated on, inter alia, numerous modifications of the preauricular and retroauricular approaches [33,35]. In cases of condylar head fracture most authors use long screws, however mini-plates and microplates may also be applied under certain conditions [33,35,36,37]. Mini-plates are a dedicated solution for the fixation of fractures of the mandibular condyle neck and condylar base [1,3,6,7,10].

Commonly, two straight mini-plates are used in fixation of the mandibular condyle base and neck fractures [18,24,25,38]. This technique serves as a reference in the evaluation of the treatment results with 3D mini-plates [7,10,18,24,25,38]. The individual shapes of 3D mini-plates are mainly compared in clinical trials to the double straight mini-plates fixation technique. Adhikari et al. assessed that the trapezoidal mini-plates can be characterized by a less challenging way of adjustment and greater speed of operation compared to the two straight mini-plates, while maintaining similar clinical treatment results [24]. The same conclusions were reached by Ahuja et al. with regard to the delta-shaped 3D mini-plates [25]. Scott et al. confirmed the superiority of trapezoidal mini-plates in terms of adaptability as well as work efficiency, and observed higher stability of bone fragments and lower displacement thereof [29]. Sukegawa et al. emphasize the need for a careful selection of the three-dimensional mini-plate shape according to the clinical situation, especially in cases of bone defects [38]. At the same time those authors report equally satisfactory treatment results with properly used 3D mini-plates compared to the double straight mini-plates technique [38].

Among the articles describing the use of 3D mini-plates, apart from those included in this analysis, one by Louvrier et al. stands out [39]. The lack of data on complications related to the osteosynthetic material did not allow us to include this work in our analysis [39]. Nevertheless, in discussing almost 500 cases of surgical access, most of which were fixated with 3D mini-plates, it is the largest study on the subject according to our knowledge [39].

Particular shapes of 3D mini-plates differ from each other in terms of biomechanics [6,13,14,33]. It has been shown that the fixation technique with one straight mini-plate does not provide sufficient stability of bone fragments [13]. The optimal arrangement of two straight mini-plates is that which transfers the forces of the condylar process’ posterior edge and the mandibular notch’s edge, consistent with the natural force balance in the undamaged mandible [6,7,10,14,17,18]. The attempts to improve treatment outcomes by using only one plate have led to the development of 3D mini-plates with an arm arrangement analogous to the above-mentioned force system [9,11].

The available 3D mini-plates shapes allow for the choice of the arrangement of holes in the proximal part along the long axis of the condyle, e.g., in a deltoidal mini-plate or perpendicularly to it, as in a trapezoidal mini-plate. Experimental studies have shown the need to apply much greater force to obtain the displacement between two elements fixed with mini-plates with 8 or more holes with screws [10]. In the material analyzed by us, the cases of using 3D mini-plates with 7 holes (lambda) and 9 holes (9-hole trapezoid) were taken into account. For these 3D mini-plates, there were no reports of osteosynthetic screw loosening, no mini-plate fracture or reoperation for any reason in the analyzed material. Nevertheless, it should be taken into account that these studies were conducted on small groups: lambda (*n* = 11), 9-hole trapezoid (*n* = 28). More than 2 holes in the distal part of the 3D mini-plate and therefore more screws distally seems to reduce the number of loose screws, as the results of our analysis suggest. A sole analysis of the most comprehensive study on 3D mini-plates within the discussed data seems to support this thesis [32]. The stability of fixation with more screws is confirmed by experimental studies [7,10,16]. In addition to increasing the number of screws in the distal bone fragment, the assessment results of mini-plates with 3 closely spaced holes in the proximal part seem promising [10].

According to our calculations, the loosening of the screws seems to be independent or only slightly dependent on the type of 3D mini-plate used. This is evidenced by the almost linear relationship between the number of fixations and the number of bolts loosening. Thus, there is a need for search and identification of other circumstances that may affect the percentage of screw loosening. The authors of the reviewed articles indicate: (1) poor bone quality; (2) bilateral condylar fractures; (3) difficulties in the correct positioning of the osteosynthetic material due to the limitations of the surgical approach; and (4) fracture line pattern, including the presence of intermediate fragments [1,28,31]. Individual dimensions of the mandibular condyles, which affect the distribution of compact and spongy bone, may also be important and may therefore imply the need to adjust the length of the screws [5]. Moreover, experimental studies show that screws may loosen as a result of mechanical overload [10].

The reasons for reoperations indicated by the authors of the analyzed articles were: (1) mispositioning of bone fragments; (2) lack of bone fragments union; (3) secondary dislocation; and (4) hematoma [26,28,32]. In view of the correlation analysis we have carried out, it is difficult to identify clear reasons for reoperation related only to the osteosynthetic material. In the data collected, we observed some indications that could suggest an association of a small number of osteosynthetic screw holes with a higher percentage of reoperations. However, there is a lack of data needed to draw firm conclusions on this subject.

Fractures of the mini-plates fixing the mandibular condyles in the technique of 2 straight mini-plates were noted in the material of Adhikari et al. and amounted to 16% of cases [24]. In turn, Hirjak et al. reported 14% of miniplate fractures using the single straight miniplate technique [40]. The paper by Hirjak et al., focused on operative access, despite the use of various fixation techniques, including 3D trapezoidal and lambda-shaped mini-plates, does not specify the number of reoperations and loosening of osteosynthetic screws and was therefore not included in our analysis [40]. Fractures of 3D mini-plates are extremely rare and three such cases were reported in the analyzed studies [27,32]. Lechler et al. describe the fracture of a 3D strut mini-plate. This fracture was most likely due to insufficient reduction of bone fragments and the subsequent overload of the 3D mini-plate, which, instead of sharing the load, had to bear it entirely [27]. The other 2 cases of fractures of 3D mini-plates concerned trapezoid and deltoid shapes and were found in the material of Zrounba et al., yet no specific data were provided [32].

In addition to the above, the authors of this paper are aware of only 2 articles mentioning clinical failure of 3D mini-plates. Sukegawa et al. describe two cases of 3D mini-platelet fractures, one of which required reoperation (lambda) and the other was detected accidentally while removing the osteosynthetic material (the shape was not given) [38]. In an article from 2007 Lauer et al. noticed loosening of osteosynthetic screws in 3 out of 16 patients treated endoscopically with the use of delta mini-plates [41]. Taking into account all the above-mentioned knowledge about the failures of 3D mini-plates, it can be suspected that the possible causes may include: (1) the incorrect plate choice depending on the type of fracture (neck or base); (2) the wrong approach depending on the type of fracture (neck or base); or (3) the incorrect choice of a plate according to the approach.

## 6. Conclusions

Randomized controlled trials regarding the use of 3D mini-plates are lacking. There is no convincing data that the number of reoperations depends on the type of 3D mini-plate used, albeit there may be an increased risk of reoperation for mini-plates with fewer holes and therefore fixed with fewer screws. The frequency of loosening of osteosynthetic screws does not seem to depend on the shape of the 3D mini-plate and probably depends on other factors. Clinical fractures of 3D mini-plates are extremely rare, in contrast to the fairly frequent fractures of straight miniplates.

## Figures and Tables

**Table 1:** Brief summary of the PICOS eligibility criteria for the systematic review [19].

	Inclusion Criteria	Exclusion Criteria
Population	Fracture in the anatomical region of the base and/or neck of the condyle of the mandible according to the AOCMF Classification System [20]	Animal patients
Intervention	Surgical treatment with 3D titanium mini-plates usage: Strut, Rhombic, Trapezoid (TCP), Delta (DCCP), Lambda, A-shape (ACP) and X-shape (XCP) patterns	Surgical treatment with a two or more mini-plates
Comparison	None or any (e.g., surgical treatment with another technique or conservative treatment)	-
Outcomes	Determination of the number of reoperations, loosening of osteosynthetic screws and the number of 3D mini-plate fractures	-
Study design	Research with a level of evidence from 1 to 3 and low risk of bias; a minimum of 10 cases in the study group	Papers published prior to 2010; non-English articles; articles non indexed in PubMed

**Table 2:** Bias risk assessment for included studies [22,23].

First Author and Year of Publication	Confounding	Selection of Participants	Classification of Intervention	Deviations of Intended Interventions	Missing Data	Measurement of Outcomes	Selection of Reported Results	Randomization Process	Overall Risk of Bias
Adhikari 2020 [24]	Not applicable	Not applicable	Not applicable	Moderate	Low	Low	Low	Low	Low (1.2)
Ahuja 2018 [25]	Not applicable	Not applicable	Not applicable	Moderate	Low	Moderate	Low	Low	Low (1.4)
Burkhard 2020 [26]	Low	Low	Low	Low	Low	Low	Low	Not applicable	Low (1.0)
Lechler 2018 [27]	Low	Low	Low	Low	Low	Moderate	Low	Not applicable	Low (1.1)
Leonhardt 2019 [28]	Low	Low	Low	Low	Low	Moderate	Low	Not applicable	Low (1.1)
Scott 2020 [29]	Not applicable	Not applicable	Not applicable	Moderate	Low	Moderate	Low	Low	Low (1.4)
Sikora 2020 [1]	Low	Low	Low	Low	Low	Moderate	Low	Not applicable	Low (1.1)
Smolka 2020 a [30]	Low	Low	Low	Low	Low	Moderate	Low	Not applicable	Low (1.1)
Smolka 2020 b [31]	Low	Low	Low	Low	Low	Moderate	Low	Not applicable	Low (1.1)
Zrounba 2014 [32]	Low	Low	Low	Low	Serious	Moderate	Low	Not applicable	Low (1.4)

**Table 3:** Individual shapes of 3D mini-plates. Original drawings by one of the authors of this paper (Z.N.).

3D Mini-Plate Shape	Trapezoid	Deltoid	Rhombus	Strut	9-Hole Trapezoid	Lambda
Drawing of a 3D mini-plate	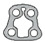	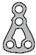	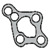	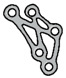	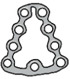	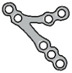

**Table 4:** Synthesis of data extracted from articles qualified for meta-analysis. Percentages greater than 5% and greater than 10% are distinguished.

First Author and Year of Publication	Level of Evidence	3D Mini-Plate Shape	Screw Holes Proximally	Screw Holes Distally	3D Mini-Plate Cases	Reoperation Cases	Screw Loosening Cases	Mini-Plate Fracture Cases
Adhikari 2020 [24]	1	Trapezoid	2	2	25	0	0	0
Ahuja 2018 [25]	1	Deltoid	2	2	10	0	0	0
Burkhard 2020 [26]	3	Deltoid	2	2	25	1 (4.0%)	0	0
		Trapezoid	2	2	18	3 (16.7%)	0	0
Lechler 2018 [27]	3	Strut	2	3	34	0	0	1 (2.9%)
Leonhardt 2019 [28]	3	Rhombus	2	3	92	6 (6.5%)	3 (3.3%)	0
Scott 2020 [29]	1	Trapezoid	2	2	22	0	0	0
Sikora 2020 [1]	2	Deltoid	2	2	90	0	3 (3.3%)	0
		Trapezoid	2	2	12	0	0	0
Smolka 2020 a [30]	3	Lambda	2	5	11	0	0	0
Smolka 2020 b [31]	3	Trapezoid	2	2	12	0	2 (16.7%)	0
Zrounba 2014 [32]	3	Trapezoid	2	2	83	0	4 (4.8%)	1 (1.2%)
		9-hole Trapezoid	4	5	28	0	0	0
		Deltoid	2	2	15	0	2 (13.3%)	1 (6.7%)
Summary	1–3	Trapezoid	2	2	150	3 (2.0%)	6 (4.0%)	1 (0.7%)
	1–3	Deltoid	2	2	140	1 (0.7%)	5 (3.6%)	1 (0.7%)
	3	Rhombus	2	3	92	6 (6.5%)	3 (3.3%)	0
	3	Strut	2	3	34	0	0	1 (2.9%)
	3	9-hole trapezoid	4	5	28	0	0	0
	3	Lambda	2	5	11	0	0	0
Total	1–3	3D mini-plates	2–4	2–5	455	10 (2.2%)	14 (3.0%)	3 (0.7%)

**Table 5:** Correlation matrix between numerical values from scientific research in a meta-analysis. Correlation values for data series where Student’s *t*-test showed *p* > 0.05 are not reported. Strong correlations, i.e., with an absolute value greater than 0.5.

Series	Screw Holes Proximally	Screw Holes Distally	Total Number of Screw Holes	3D Mini-Plate Cases	Reoperation Cases	Screw Loosening Cases	Screw Loosening Rate	Mini-Plate Fracture Cases
Screw holes proximally	*p* > 0.05							
Screw holes distally	*p* > 0.05	*p* > 0.05						
Total number of screw holes	0.83	0.96	*p* > 0.05					
3D mini-plate cases	−0.06	−0.07	−0.07	*p* > 0.05				
Reoperation cases	*p* > 0.05	*p* > 0.05	*p* > 0.05	*p* > 0.05	*p* > 0.05			
Screw loosening cases	−0.20	−0.24	−0.25	0.79	*p* > 0.05	*p* > 0.05		
Screw loosening rate	−0.16	−0.27	−0.25	−0.01	*p* > 0.05	0.56	*p* > 0.05	
Mini-plate fracture cases	−0.14	−0.12	−0.14	0.18	*p* > 0.05	0.37	*p* > 0.05	*p* > 0.05

**Table 6:** Correlation matrix between summarized numerical values for each 3D mini plate shape included in the meta-analysis. Correlation values for data series where Student’s *t*-test showed *p* > 0.05 are not reported. Strong correlations, i.e., with an absolute value greater than 0.5.

	Screw Holes Proximally	Screw Holes Distally	Total Number of Screw Holes	3D Mini-Plate Cases	Reoperation Cases	Screw Loosening Cases	Screw Loosening Rate	Mini-Plate Fracture Cases
Screw holes proximally	*p* > 0.05							
Screw holes distally	*p* > 0.05	*p* > 0.05						
Total number of screw holes	0.83	0.94	*p* > 0.05					
3D mini-plate cases	−0.39	−0.88	−0.78	*p* > 0.05				
Reoperation cases	*p* > 0.05	*p* > 0.05	−0.45	0.53	*p* > 0.05			
Screw loosening cases	*p* > 0.05	*p* > 0.05	−0.76	0.99	*p* > 0.05	*p* > 0.05		
Screw loosening rate	−0.44	−0.82	−0.76	0.97	*p* > 0.05	0.97	*p* > 0.05	
Mini-plate fracture cases	−0.45	−0.80	−0.74	0.59	*p* > 0.05	*p* > 0.05	0.40	*p* > 0.05

## Data Availability

Data sharing not applicable. No new data were created or analyzed in this study. Data sharing is not applicable to this article.
